# Beyond Size: Advanced MRI Breakthroughs in Predicting Intracranial Aneurysm Rupture Risk

**DOI:** 10.3390/jcm14093158

**Published:** 2025-05-02

**Authors:** Jose E. Leon-Rojas

**Affiliations:** Grupo de Investigación Cerebro, Emoción y Conducta (CEC), Facultad de Medicina, Universidad de las Américas (UDLA), Quito EC170516, Ecuador; jose.leon.rojas@udla.edu.ec

**Keywords:** intracranial aneurysm, rupture risk, magnetic resonance imaging, vessel wall imaging, 4D flow MRI, quantitative susceptibility mapping, machine learning

## Abstract

Intracranial aneurysms (IAs) are present in approximately 3–5% of the global population and carry a significant risk of rupture, leading to subarachnoid haemorrhage (SAH), a condition associated with high morbidity and mortality. Even with developments in neuroimaging, fundamental clinical difficulty remains in precisely predicting which aneurysms will rupture. Although aneurysm size, location, and patient history define traditional risk assessment, these elements by themselves have insufficient predictive ability. Key elements in rupture risk are aneurysm wall biology, haemodynamics, and inflammation; recent developments in magnetic resonance imaging (MRI) including high-resolution vascular wall imaging (VWI), 4D flow MRI, and quantitative susceptibility mapping (QSM) provide fresh insights on these aspects. The present evidence on these sophisticated MRI techniques is synthesised in this review of the literature, which also analyses their clinical relevance and addresses newly developed computational methods like machine learning for better risk stratification. I underline important studies showing the diagnostic and prognostic worth of MRI-based biomarkers, discuss present constraints, and suggest future lines of research. Personalised aneurysm care could benefit from the combination of multiparametric MRI data with artificial intelligence (AI), hence improving patient outcomes.

## 1. Introduction

Affecting an estimated 3–5% of the worldwide population, intracranial aneurysms (IAs) are pathogenic dilations of cerebral arteries that are more common in women and older individuals [1,2], with an overall prevalence of 3.2% (95% CI, 1.9–5.2) in populations without comorbidity [1]. This prevalence increases in those with autosomal dominant polycystic kidney disease (ADPKD) and with a positive family history of IA to 6.9% (95% CI, 3.5–14%) and 3.4% (95% CI, 1.9–5.9%), respectively [1]. Many IAs remain asymptomatic, but their rupture causes subarachnoid haemorrhage (SAH), a terrible condition with a death rate of more than 35% and major long-term disability among survivors [3]; furthermore, a cost-effectiveness study from Switzerland reported that the total cost per patient with SAH reached EUR 344,277 (95% CI, 268,383–420,171), showcasing SAH’s significant economic weight [4]. Timely intervention depends on precise identification of an IA at danger of rupture in order to avoid all the aforementioned calamities.

**Figure 1:**
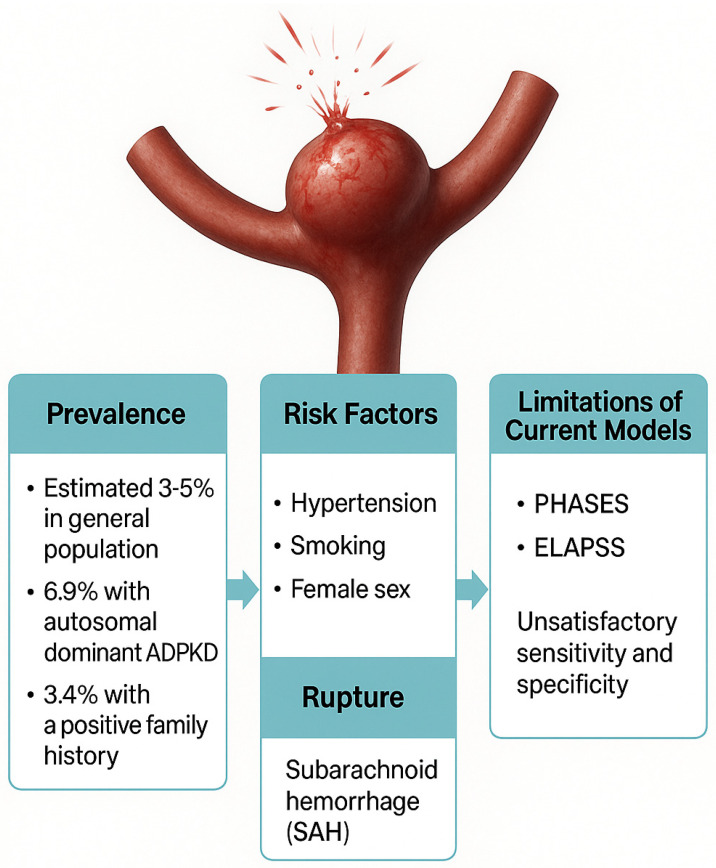
Summary of the important characteristics of an intracranial aneurysm.

Current recommendations for IA care depend on the IA’s size, location, and patient-specific variables including family history, hypertension, and smoking [5]. Although they integrate these criteria, widely used risk stratification tools such as the PHASES and ELAPSS scores have unsatisfactory sensitivity and specificity [6,7,8]. For instance, the sensitivity and specificity to discriminate an unruptured IA from SAH using a PHASES score threshold of 4 has been reported to be 46% and 84%, respectively, with an AUC of 0.66 (95% CI, 0.62 to 0.70) [7]. Additionally, a notable fraction of ruptured IAs are smaller than the conventional 7 mm cut-off, therefore highlighting the shortcomings of size-based evaluation by itself [9]. Finally, morphological features (e.g., aspect ratio, irregular shape) and hemodynamic factors (e.g., wall shear stress) derived from computational fluid dynamics (CFD) have been explored but face challenges in clinical translation due to variability in measurement techniques and a lack of standardised thresholds [10]. A graphical summary of important characteristics of IAs is provided in Figure 1.

New biomarkers for rupture risk have come from recent developments in magnetic resonance imaging (MRI) technology, allowing for non-invasive evaluation of IA wall biology and haemodynamics. While 4D flow MRI offers dynamic blood flow measurements without regard to CFD assumptions, high-resolution vascular wall imaging (VWI) detects inflammatory alterations and wall weakening [11,12]. By pointing out intra-aneurysmal thrombus development, a possible indicator of instability, quantitative susceptibility mapping (QSM) enhances these methods even more [13]. These modalities, together with machine learning (ML) techniques, enable a more complete assessment of an IA pathogenesis. Therefore, the objectives of this review are to (1) consolidate the most recent information on sophisticated MRI approaches for IA rupture risk prediction; (2) analyse the clinical relevance and restrictions of these techniques; (3) discuss how multiparametric MRI combined with artificial intelligence might help to better stratify IA rupture risk; and (4) suggest future study paths to improve clinical utility and predictive accuracy.

A structured literature search was performed to identify studies relevant to advanced MRI techniques for the prediction of IA rupture risk. The search was conducted in PubMed/MEDLINE, Scopus, and Web of Science, from January 2000 to March 2025 for articles written in both English and Spanish. The search terms used were “intracranial aneurysm” OR “cerebral aneurysm”) AND (“rupture risk” OR “rupture prediction” OR “diagnosis”) AND (“MRI” OR “magnetic resonance imaging” OR “vessel wall imaging” OR “VWI” OR “4D flow MRI” OR “quantitative susceptibility mapping” OR “QSM” OR “high-resolution MRI” OR “artificial intelligence” OR “machine learning” OR “deep learning”. I included original research articles, systematic reviews, and meta-analyses focusing on advanced MRI techniques (VWI, 4D flow MRI, QSM) in the evaluation of intracranial aneurysm stability or rupture risk as well as studies employing AI/machine learning models using MRI-derived data for IA rupture risk assessment; conference abstracts, editorial comments, and letters to the editor as well as studies not including advanced MRI methods or not addressing rupture prediction were not included. In total, around 1800 articles resulted from the search and 40 were selected based on relevance and a direct relationship to reporting advanced MRI techniques. The research was conducted in accordance with the design as described in the Supplementary Material.

## 2. Current Challenges in Intracranial Aneurysm Rupture Risk Prediction

The dependence on traditional risk factors for cerebral aneurysm rupture prediction offers numerous important constraints that affect clinical decision-making. With the commonly reported 7 mm threshold determined from the International Study of Unruptured Intracranial Aneurysms (ISUIA), traditional models mostly stress IA size as the main driver of rupture risk [14]. Nevertheless, later investigations have repeatedly shown that this size criterion is insufficiently sensitive since several studies find that an important percentage of burst IAs are less than 7 mm in maximum diameter [9]. For instance, a prospective Finnish cohort study reported 96 participants with IA of less than 7 mm, of which 25% ended up having SAH [15]. This disparity draws attention to a crucial flaw in size-based risk assessment that can cause patients with smaller but high-risk IAs to lose possible chances for preventative intervention. While statistically significant in population studies, patient-specific elements included in current scoring systems (hypertension, smoking history, and family history of aneurysmal subarachnoid haemorrhage) show low prognostic value at the individual patient level [16,17]. Among the most often used risk assessment systems, the PHASES score combines these elements but shows only limited discrimination for the risk of rupture in smaller IAs (AUC = 0.634) [17]. The inability of the score to consider important biological elements such wall inflammation or haemodynamic stress patterns that can induce IA instability independent of conventional risk variables results in this restricted predictive performance [11].

Conventional risk variables’ time dynamics impose more restrictions on applicability and predictive power. Most current models offer stationary risk evaluations devoid of consideration for any changes in IA form or patient risk profile over time [18]. Longitudinal investigations have shown that IAs may remain stable for years before experiencing fast morphological changes prior to rupture, a phenomena not adequately reflected by existing risk assessment paradigms [19,20,21]. This dynamic feature of IA behaviour emphasises the need for more advanced monitoring techniques beyond regular size measures. Most fundamentally, traditional risk factors concentrate mostly on the likelihood of rupture without giving enough thought to the biological processes behind IA instability. Their capacity to differentiate between stable and unstable IAs of similar size and location is limited by this mechanistic gap. Unstable IAs show unique histological traits including inflammatory infiltrates, neovascularisation, and wall weakening, which are traits not reflected in standard risk assessment models according to pathology studies [22]. These limits of traditional risk variables have motivated the hunt for more complex biomarkers of IA instability, especially those obtained from new imaging technologies that can probe the underlying biology of an IA advancement. Such new biomarkers combined with conventional risk factors should provide better risk categorisation and solve the inadequacies of present models [23].

## 3. Advanced MRI Techniques for Rupture Risk Assessment

**Figure 2:**
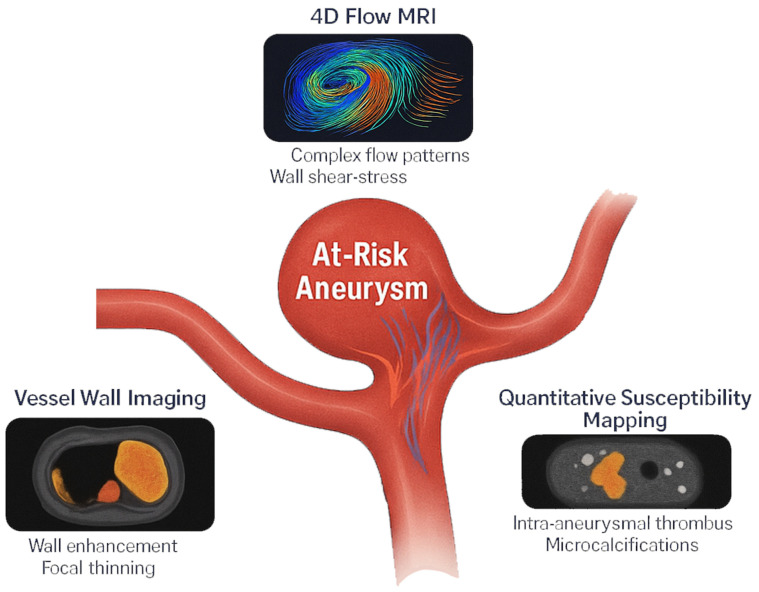
Advanced MRI techniques for intracranial aneurysm assessment.

Advanced MRI techniques useful for assessing IA status and predicting rupture risk include vessel wall imaging (VWI), 4D Flow MRI, and quantitative susceptibility mapping (QSM) and are depicted in Figure 2.

### 3.1. High-Resolution Vessel Wall Imaging (VWI)

For the evaluation of cerebral IAs, high-resolution vessel wall imaging (VWI) has become a revolutionary method allowing for direct visualisation of vessel wall disease, something that was impossible before with non-invasive imaging. By achieving spatial resolutions of 0.4–0.6 mm isotropic at 3T and sub-0.4 mm at 7T, modern VWI techniques enable identification of minor wall anomalies that can correlate with IA rupture risk [24,25]. In a study of 108 IAs, the finding of circumferential aneurysmal wall enhancement (CAWE) was used for the detection of symptomatic or ruptured IAs (i.e., unstable IAs); multivariable logistic regression showed that CAWE was the only variable associated with unstable IAs with an odds ratio of 9.20 (95% CI, 2.92–29.0; *p* = 0.0002) and that CAWE was not related to aneurysmal size [26]. Certainly, aneurysmal wall enhancement (AWE) is a promising VWI biomarker that, together with wall thickness variability analysis and multi-contrast VWI signatures, has shown promise in rupture risk assessment.

Quantitative analysis of AWE has revealed that these enhanced areas show 2.3–3.1 higher signal intensity [27] and represent regions of neovascularization within the vessel wall (vasa vasorum) and infiltration of granulocytes (MPO+) and macrophages, showcasing the instability of the IA [23,28]. A 2018 systematic review and meta-analysis, including a total of 505 saccular IAs, reported that AWE was associated with higher odds of detecting an unstable IA with an OR of 20.0 (95% CI, 6.4–62.1); furthermore, the authors found a pooled sensitivity of 95.0%, specificity of 62.7%, positive predictive value of 55.8%, and negative predictive value of 96.2% for detecting unstable IAs [29].

Another important biomarker of VWI is the measurement of the variability of the vessel’s wall thickness. 7T MRI studies have demonstrated that imaging the wall’s thickness is possible and has good correlation with ex vivo histological analysis [30]. Furthermore, a 7T turbo spin echo (TSE)-based vessel wall MR sequence study with a 0.8 mm isotropic resolution reported that spatial wall thickness variation was inversely correlated with shear stress of the IA wall (i.e., the thinner the vessel wall the greater the wall shear stress and, consequently, the risk of rupture) [31]. Finally, the utilisation of different MR contrasts (i.e., T1 and T2 signals) can provide valuable information to generate vessel wall signatures with high propensity of rupture. For instance, in a 3D-T1 weighted fast spin echo (FSE) 3T MR sequence, the wall enhancement index (WEI) can be calculated by obtaining the ratio between the signal intensity (SI) of the IA wall against the SI of normal brain tissue before and after contrast enhancement [32]. A study of 104 IAs (28 ruptured) reported that WEI was significantly higher in ruptured (1.70 ± 1.06 vs. 0.89 ± 0.88) vs. unruptured IAs (*p* = 0.0001); the optimal cut-off value of the WEI was calculated to be 0.53 to differentiate ruptured from unruptured IAs, yielding a sensitivity of 96% and specificity of 47% (with an AUC of 0.75) [32]. Furthermore, 3D mapping of aneurysmal wall enhancements has proven to be strongly correlated with other determinants of aneurysmal instability such as size, size ratio, and aspect ratio (*p* < 0.05) [33].

Despite the promise of high-resolution VWI, several challenges lie ahead. There is a lack of imaging protocol standardisation, and recommendations from expert consensus have been published by the American Society of Neuroradiology to mitigate this [34]. The main technical specifications for the proper realisation of VWI include the following: (1) high spatial resolution; (2) multiplanar two-dimensional or three-dimensional acquisitions; (3) various tissue weightings; and (4) suppression of signals in luminal blood and cerebrospinal fluid [34]. To obtain high-fidelity VWI, careful selection and implementation of specific MRI sequences and parameters is paramount. The sequences most frequently employed include 3D turbo spin echo techniques, specifically SPACE (Sampling Perfection with Application optimised Contrasts using different flip angle Evolutions), VISTA (Volumetric Isotropic Turbo Spin Echo Acquisition), and CUBE, which achieve good isotropic resolution while preserving an adequate signal-to-noise ratio [24,25]. Black-blood imaging remains a central element; methods that suppress luminal signal, such as those utilising variable flip angles in conjunction with double inversion recovery or motion-sensitised driven equilibrium, serve to enhance delineation of the vessel wall [24,25,34]. Post-contrast VWI, undertaken approximately 4–6 min following gadolinium administration, relies on T1-weighted black-blood sequences and plays a pivotal role in assessing aneurysmal wall enhancement. Furthermore, the application of multiple contrast weightings (including T1, T2, and proton-density) has proven beneficial for exploring wall composition and inflammatory changes [24,25,34]. To improve reproducibility across institutions, protocols must also incorporate cerebrospinal fluid suppression, robust fat saturation, and motion correction strategies [34]. Another important challenge is the lack of longitudinal data regarding VWI changes in IAs for rupture prediction; this is currently limiting this very promising technique. Finally, another important constraint, especially in resource-limited settings, is the use of 7T MR imaging, which has been shown to have better results than 3T VWI; the latter tends to overestimate wall thickness [35].

### 3.2. Four-Dimensional Flow MRI for Hemodynamic Assessment

By means of thorough, time-resolved three-dimensional blood flow measurements across the cardiac cycle, four-dimensional flow magnetic resonance imaging (4D flow MRI) has transformed the haemodynamic evaluation of intracranial IAs [36]. It employs dedicated time-resolved, three-dimensional phase-contrast sequences with three-directional velocity encoding, designed to capture dynamic cerebrovascular haemodynamics across the entire cardiac cycle [36,37]. Central to its accuracy is the careful calibration of velocity encoding (VENC) thresholds, which are typically set between 100 and 150 cm/s for intracranial arteries to reduce phase aliasing and ensure reliable quantification. Spatial resolution ranges from 0.6 to 1.0 mm isotropic resolution, with temporal resolution typically between 20 and 40 ms; these parameters strike a balance between anatomical fidelity and temporal acuity [12,36,37,38]. Acquisition durations vary from 7 to 12 min and can be modulated by advanced acceleration strategies such as parallel imaging or compressed sensing [12,36,37,38]. Retrospective cardiac gating is routinely incorporated, often accompanied by respiratory compensation, to mitigate motion-related artefacts and ensure consistent signal across the cardiac cycle [12,36,37,38]. Subsequent data reconstruction relies on specialised post-processing platforms (i.e., GTFlow, Arterys, or bespoke institutional software), enabling detailed analysis of complex flow patterns free from the geometric simplifications needed by CFD simulations [12,36,37,38]. New understanding of the haemodynamic parameters related to IA instability and rupture risk has come from the clinical application of 4D flow MRI [12,38]. This technique provides multiple haemodynamic biomarkers that are useful in the analysis of an intracranial IA, such as (1) wall shear stress (WSS), which is the stress created in the wall by the friction and shear produced by the tangential viscosity of the blood within the vessel or aneurysm; (2) pulse wave velocity (PWV), which is the speed at which the pulse wave travels along the vessel; (3) kinetic energy (KE), which is the energy that exists in the flow of blood; (4) turbulent kinetic energy, which is the amount of energy lost due to disturbed or turbulent blood flow; and (5) the oscillatory shear index (OSI), which is how much the WSS changes direction during a heartbeat [39].

Several important haemodynamic characteristics have been found by recent 4D flow MRI investigations differentiating stable IAs from unstable IAs [12]. A study looking at 37 unruptured IAs compared six 3D morphology proxy parameters of IA instability (major axis length, volume, surface area, flatness, shape index, and curvedness) with the results of 4D flow MRI; they reported that time-averaged WSS correlated negatively with volume and flatness, peak-systolic WSS and time-averaged WSS correlated positively with shape index, and OSI correlated negatively with shape index but positively with flatness [40].

Ruptured aneurysms routinely show more complicated flow patterns with high-speed blood jets originating from the parent vessel and hitting the opposite aneurysm wall, while unruptured aneurysms reveal a more constant flow pattern characterised by a big vortex that remains constant during the cardiac cycle [41]. Furthermore, complex Newtonian and Non-Newtonian CFD simulations have shown that ruptured aneurysms exhibit high-frequency flow fluctuations within the aneurysm sac, but these models are affected by resolution, which could be solved by the use of 7T 4D flow MRI [41,42]. Other than characterising the flow characteristics of ruptured vs. unruptured aneurysms, this technique is useful for differentiating stable vs. unstable aneurysms. A study that included 213 IAs (with 64 unstable IAs) using 3T 4D flow MRI, with a 3D black-blood T1-weighted volumetric isotropic turbo spin echo acquisition (T1-VISTA), crafted a hybrid model by combining 4D flow MRI and the Multi-crop Attention Branch Network (MicroAB-Net), an artificial intelligence deep learning model looking at aneurysmal size [12]. They reported significant differences in multiple haemodynamic parameters between stable and unstable aneurysms, including differences in minimum velocity (V) of 15.87 ± 10.92 cm/s for unstable IAs versus 20.27 ± 11.8 cm/s for stable IAs (*p* = 0.017); average V of 28.54 ± 13.26 cm/s for unstable IAs versus 33.49 ± 14.86 cm/s for stable IAs (*p* = 0.026); maximum V of 42.43 ± 17.49 cm/s for unstable IAs versus 50.94 ± 21.61 cm/s for stable IAs (*p* = 0.009); average flow of 1.82 ± 1.68 mL/s for unstable IAs versus 1.53 ± 1.75 mL/s for stable IAs (*p* = 0.048); maximum flow of 3.53 ± 3.30 mL/s for unstable IAs versus 2.69 ± 2.83 mL/s for stable IAs (*p* = 0.012); maximum WSS of 0.39 ± 0.40 N/m^2^ for unstable IAs versus 0.76 ± 0.65 N/m^2^ for stable IAs (*p* < 0.001); and time averaged WSS of 0.22 ± 0.28 N/m^2^ for unstable IAs versus 0.44 ± 0.32 N/m^2^ for stable IAs (*p* < 0.001) [12]. Furthermore, when evaluating the diagnostic capabilities of their hybrid model and comparing it with logistic regression models that used well-known aneurysm rupture prediction scores such as PHASES and ELAPSS, they found that their model was the best performing, with an AUC of 0.854 (95% CI, 0.791–0.916), sensitivity of 73.4% (95% CI, 61.4–82.7), and specificity of 85.2% (95% CI, 78.6–90.1); in contrast, the PHASES model had an AUC of 0.725 (95% CI, 0.646–0.803), sensitivity of 75.0% (95% CI, 63.0–84.0), and specificity of 62.4% (95% CI, 54.4–69.8) [12]. Even when looking at 4D flow MRI by itself, it outperformed all other models except for the hybrid one with an AUC of 0.809 (95% CI, 0.740–0.877), sensitivity of 68.2% (95% CI, 56.1–78.1), and specificity of 83.0% (95% CI, 76.0–88.2), showcasing that the diagnostic power of 4D flow MRI appears to be higher than conventionally used stratification IA scores [12].

Another important application of 4D flow MRI can be the evaluation of flow stagnation patterns in IAs, particularly quantitative metrics like total/relative residence times (i.e., the time blood spends within the IA’s sac), and stagnation volume ratio [43]. These metrics have been previously assessed by simulations or in vivo studies. For instance, a study looked into surgical video recordings of unruptured IAs and used CFD to calculate the relative residence time; the authors found that a prolonged relative residence time (RT) was associated with the presence of an atherosclerotic lesion within the IA [44]. These flow stagnation parameters could be calculated using 4D flow MRI, and more studies are required in order to assess their utility. So far, feasibility studies have shown that the calculation of RT in 4D flow MRI is possible and its results correlate with other relevant haemodynamic metrics [43]. Certainly, good agreement with CFD for important hemodynamic parameters has validated the dependability of 4D flow MRI measurements [45,46]. Studies have even proposed that 4D flow MRI, if merged with CFD models, could overcome some of its inherent limitations such as acquisition noise, flow artifacts, and resolution constraints [45].

### 3.3. Quantitative Susceptibility Mapping (QSM)

Particularly for the detection of intra-aneurysmal thrombus development and wall calcification, quantitative susceptibility mapping (QSM) could become a powerful method for assessing IA pathophysiology by quantifying magnetic susceptibility changes in tissues [47]. This method generates three-dimensional susceptibility maps allowing for exact identification of paramagnetic compounds including iron-containing haemoglobin breakdown products in thrombi or sentinel microbleeds [13,47]. It uses multi-echo gradient echo (GRE) sequences, optimised to capture minute magnetic susceptibility variations and map tissue composition in a non-contrast-enhanced manner [13,47]. High field strengths are employed to maximise susceptibility contrast, while the GRE sequence typically includes at least five echoes, with echo times spaced by approximately 5–7 ms to ensure adequate sampling of phase evolution [13,47]. Acquisitions are performed at an isotropic spatial resolution of around 1 mm, with a wide receiver bandwidth to mitigate chemical shift artefacts; the post-processing workflow is intricate, beginning with phase unwrapping to correct for discontinuities, followed by background field removal (often executed using algorithms such as SHARP or its variable-kernel derivative V-SHARP) and culminating in susceptibility map reconstruction via dipole inversion, commonly using techniques like MEDI or iLSQR [13,47]. A major advantage of QSM lies in its non-reliance on contrast agents, rendering it particularly well-suited for longitudinal studies; however, its clinical implementation remains constrained by technical variability across scanners and field strengths, and the absence of standardised acquisition and reconstruction protocols continues to limit its broader adoption in intracranial aneurysm research.

QSM’s clinical value stems from its capacity to non-invasively characterise aneurysm wall composition, therefore providing insights into biological processes connected to rupture risk unnoticed by traditional imaging modalities [47]. However, QSM has been scarcely used in research for IA detection or rupture risk prediction; it has been used, however, for other scenarios such as dissecting intracranial hematomas and atherosclerotic calcifications, quantifying cerebral iron in ageing individuals, and correlating cortical thickness and network connectivity in Alzheimer patients, and it has been able to characterise relevant cardiovascular abnormalities (i.e., blood oxygenation measurement, myocardial iron content estimation, myocardial fibre orientation, and carotid plaque composition) [48,49,50,51].

When looking at the application of QSM in IAs, I only found articles reporting its utility in the detection of microbleeds associated with sentinel headaches, reaching up to 100% sensitivity and specificity [13,52,53]. However, there is a significant scarcity of research regarding QSM and IAs looking into other factors of aneurysmal pathology or risk stratification (i.e., a PubMed search yielded a total of 13 results, of which only 3 were related with IAs—all of which were related to sentinel bleeds). This points towards a massive research gap, as QSM has all the theoretical potential of being a useful imaging biomarker in the assessment of IAs.

Table 1 provides a summarised comparison between the three aforementioned advanced MRI techniques.

## 4. Integration of Artificial Intelligence with MRI for IAs

By enabling automated analysis of challenging imaging biomarkers, the combination of machine learning (ML) with sophisticated MRI technologies has greatly improved intracranial aneurysm rupture risk prediction. In particular, convolutional neural networks (CNNs), deep learning algorithms, have shown exceptional ability to extract tiny patterns from high-dimensional MRI data that evade traditional radiologic evaluation. For example, a study using a 3-dimensional convolutional neural network architecture (HeadXNet) analysed a dataset containing a total of 818 examinations from 662 patients (with an aneurysm in 40.1% of them); they showed that by using the CNN, the clinician’s mean sensitivity increased by 0.059 (95% CI, 0.028–0.091; *p* = 0.01), and the mean accuracy increased by 0.038 (95% CI, 0.014–0.062; *p* = 0.02) [54]. Another study using a CNN in 3D time-of-flight MRI reported an overall sensitivity of 90% (96% for IAs between 3 and 7 mm and 100% for those > 7 mm) [55].

Artificial intelligence (AI) has also been used to aid in rupture risk prediction of IAs. A study that included 594 anterior communicating artery aneurysms used a two-layer feed-forward artificial neural network trained with 17 parameters (including aneurysmal morphological characteristics, demographic data, and hypertension and smoking history) [56]. They reported an AUC of 0.928 and an overall rupture prediction accuracy of 94.8% [56]. Certainly, multiple studies have looked at the use of AI in aneurysm detection or risk prediction by using datasets originating from computed tomography angiography, magnetic resonance imaging angiography, 3D time-of-flight MRI, and digital subtraction angiography [57]. However, these AI models still need to integrate the parameters discussed within this article and originating from more advanced MRI methods such as VWI and 4D flow MRI; achieving this could potentiate the diagnostic accuracy and risk prediction of models assisted by AI even more. Several ML techniques have already been used for cardiac 4D flow MRI and have aided in improving the constraints of the method or in the identification of relevant structures [58].

Future cerebral aneurysm management could benefit a lot from the combination of AI with cutting-edge MRI methods such 4D flow MRI and VWI. AI could find minor trends in aneurysm wall structure, inflammation, and blood flow dynamics that now defy conventional diagnostic frameworks by aggregating complicated biological and haemodynamic data. By analysing dynamic signs of instability (such as changing wall enhancement or turbulent flow signatures), machine learning algorithms may help us go beyond static morphological examinations to forecast rupture risk with unheard-of accuracy. These technologies could combine multimodal imaging data with patient-specific variables to create individualised risk profiles, therefore directing choices about monitoring schedules or preventative treatments. Although issues such as standardising imaging techniques and model interpretability still exist, developments in artificial intelligence could soon aid in building confidence in algorithmic forecasts. In the end, this interplay of artificial intelligence and sophisticated MRI data could shift aneurysm treatment towards proactive, customised approaches, spotting high-risk lesions early and maximising treatment timing to enhance patient outcomes and lower unnecessary surgical interventions.

## 5. Future Directions and Limitations

Future research should focus on the integration of sophisticated MRI biomarkers with machine learning (ML) in order to improve IA risk classification and create strong, generalisable predictive models. Prospective multicentre studies are required to evaluate the clinical relevance of VWI biomarkers, such aneurysmal wall enhancement (AWE) and wall thickness variability, in various populations. Efforts on standardising haemodynamic parameters (e.g., wall shear stress, oscillatory shear index) across institutions and improving turbulence modelling to increase accuracy in tiny aneurysms should be the focus of new 4D flow MRI studies. Research on QSM is still lacking; studies have to assess its ability to identify microcalcifications and intra-aneurysmal thrombus development, which might be new rupture risk predictors. Furthermore, long-term studies with appropriate follow-up are necessary for identifying pre-rupture pathophysiological changes by tracking MRI biomarker progression such as AWE patterns or haemodynamic shifts. Ultimately, clinical acceptance depends on cost-effectiveness studies contrasting improved MRI techniques with current norms, especially in resource-constrained settings. Finally, AI systems could be developed to include genetic and proteomic biomarkers in addition to multimodal MRI data (VWI, 4D flow, QSM), therefore enabling dynamic risk prediction models including temporal changes in aneurysm biology.

Although advanced MRI methods show promise, important limitations may prevent their broad clinical application. VWI is still limited by the use of inconsistent imaging protocols across institutions and scientific studies; AWE quantification is affected by spatial resolution and contrast timing differences. Although 3T systems may overstate dimensions, high-field (7T) MRI is inaccessible in most centres even if it is better for wall thickness measurements. While longer acquisition times enhance motion artefact risks, poor spatial resolution can obscure haemodynamic features in small aneurysms (<3 mm) for 4D flow MRI. Finally, challenges in artificial intelligence integration include “black box” model opacity, dataset bias towards larger aneurysms or aneurysms in specific locations, and inadequate representation of unusual phenotypes (e.g., fusiform aneurysms) in training data. All of the methods discussed in this manuscript have cost and accessibility restrictions; advanced MRI techniques need specialised gear and knowledge not found outside tertiary centres and scarce in developing nations. Dealing with these constraints will require group initiatives to harmonise imaging techniques, validate biomarkers in prospective cohorts, and provide economical resources for the acquisition of these advanced techniques.

## 6. Conclusions

Advanced MRI techniques, including vessel wall imaging, 4D flow MRI, and quantitative susceptibility mapping, offer transformative potential for intracranial aneurysm risk stratification by providing biomarkers of wall inflammation, hemodynamic stress, and thrombus formation that traditional metrics cannot capture fully. While these methods address critical gaps in size-based assessment, challenges such as protocol standardisation, accessibility of high-field systems, and AI model interpretability must be resolved to translate research findings into clinical practice. To enable dynamic, customised risk prediction, future efforts should give multicentre validation of these MRI biomarkers top priority, in addition to the development of affordable imaging techniques and the integration of machine learning data with multimodal data. Harnessing these tools fully will depend on cooperative innovation across radiology, neurosurgery, and computational science so early detection of high-risk aneurysms and optimal therapeutic techniques would result in significant benefit for our patients.

## Figures and Tables

**Table 1:** Relevant characteristics of VWI, 4D flow MRI, and QSM in aneurysm evaluation.

Feature	Vessel Wall Imaging (VWI)	4D Flow MRI	Quantitative Susceptibility Mapping (QSM)
Primary Application	Wall inflammation, neovascularization, structural integrity	Hemodynamic stress, flow patterns, wall shear stress dynamics	Thrombus iron content, microcalcifications, wall composition, sentinel bleeding
Key Biomarkers	- Aneurysmal wall enhancement (AWE) - Wall thickness variability	- Wall shear stress (WSS) - Oscillatory shear index - Flow stagnation - Flow velocity - Kinetic energy	- Susceptibility values - Iron quantification
Advantages	- Direct wall pathology visualisation - High sensitivity for inflammation	- Real-time flow dynamics without CFD assumptions	- Detects sentinel bleeding with up to 100% sensitivity and specificity
Limitations	- Requires high-field MRI for best results - Protocol standardisation needed	- Limited resolution for small aneurysms (<3 mm) - Long acquisition times	- Not validated for IAs - Inter-scanner susceptibility variability
Clinical Utility	Rupture risk prediction (AWE: 95% sensitivity, 63% specificity)	Identifies high-risk haemodynamics (AUC = 0.85 vs. PHASES AUC = 0.73)	- Has only been used for sentinel bleeding
Cost/Accessibility	High (7T systems rare; 3T requires expertise)	Moderate (requires specialised 4D flow software)	Low (postprocessing-dependent; no contrast needed)

## Supplementary Materials

The following supporting information can be downloaded at: https://www.mdpi.com/article/10.3390/jcm14093158/s1.

## Abbreviations

The following abbreviations are used in this manuscript:

AUC	Area Under the Curve
AI	Artificial Intelligence
AWE	Aneurysmal Wall Enhancement
CNN	Convolutional Neural Network
CFD	Computational Fluid Dynamics
ELAPSS	Earlier SAH, Location, Age, Population, Size, and Shape (risk score)
IA	Intracranial Aneurysm
ISUIA	International Study of Unruptured Intracranial Aneurysms
KE	Kinetic Energy
ML	Machine Learning
MPO	Myeloperoxidase
MRI	Magnetic Resonance Imaging
OSI	Oscillatory Shear Index
PHASES	Population, Hypertension, Age, Size of aneurysm, Earlier SAH, Site (risk score)
PWV	Pulse Wave Velocity
QSM	Quantitative Susceptibility Mapping
RT	Residence Time
SI	Signal Intensity
T1-VISTA	T1-weighted Volumetric Isotropic Turbo spin echo Acquisition
TOF-MRA	Time-of-Flight Magnetic Resonance Angiography
TSE	Turbo Spin Echo
VWI	Vessel Wall Imaging
WEI	Wall Enhancement Index
WSS	Wall Shear Stress

## References

[1] Vlak M.H., Algra A., Brandenburg R., Rinkel G.J. (2011). Prevalence of Unruptured Intracranial Aneurysms, with Emphasis on Sex, Age, Comorbidity, Country, and Time Period: A Systematic Review and Meta-Analysis. Lancet Neurol..

[2] Hishikawa T., Date I. (2017). Unruptured Cerebral Aneurysms in Elderly Patients. Neurol. Med. Chir..

[3] Etminan N., Rinkel G.J. (2016). Unruptured Intracranial Aneurysms: Development, Rupture and Preventive Management. Nat. Rev. Neurol..

[4] Seule M., Oswald D., Muroi C., Brandi G., Keller E. (2020). Outcome, Return to Work and Health-Related Costs After Aneurysmal Subarachnoid Hemorrhage. Neurocritical Care.

[5] Thompson B.G., Brown R.D., Amin-Hanjani S., Broderick J.P., Cockroft K.M., Connolly E.S., Duckwiler G.R., Harris C.C., Howard V.J., Johnston S.C. (2015). Guidelines for the Management of Patients With Unruptured Intracranial Aneurysms: A Guideline for Healthcare Professionals From the American Heart Association/American Stroke Association. Stroke.

[6] Greving J.P., Wermer M.J.H., Brown R.D., Morita A., Juvela S., Yonekura M., Ishibashi T., Torner J.C., Nakayama T., Rinkel G.J.E. (2014). Development of the PHASES Score for Prediction of Risk of Rupture of Intracranial Aneurysms: A Pooled Analysis of Six Prospective Cohort Studies. Lancet Neurol..

[7] Bijlenga P., Gondar R., Schilling S., Morel S., Hirsch S., Cuony J., Corniola M.-V., Perren F., Rüfenacht D., Schaller K. (2017). PHASES Score for the Management of Intracranial Aneurysm: A Cross-Sectional Population-Based Retrospective Study. Stroke.

[8] Backes D., Rinkel G.J.E., Greving J.P., Velthuis B.K., Murayama Y., Takao H., Ishibashi T., Igase M., terBrugge K.G., Agid R. (2017). ELAPSS Score for Prediction of Risk of Growth of Unruptured Intracranial Aneurysms. Neurology.

[9] Backes D., Rinkel G.J.E., Laban K.G., Algra A., Vergouwen M.D.I. (2016). Patient-and Aneurysm-Specific Risk Factors for Intracranial Aneurysm Growth: A Systematic Review and Meta-Analysis. Stroke.

[10] Xiang J., Natarajan S.K., Tremmel M., Ma D., Mocco J., Hopkins L.N., Siddiqui A.H., Levy E.I., Meng H. (2011). Hemodynamic–Morphologic Discriminants for Intracranial Aneurysm Rupture. Stroke.

[11] Larsen N., Flüh C., Saalfeld S., Voß S., Hille G., Trick D., Wodarg F., Synowitz M., Jansen O., Berg P. (2020). Multimodal Validation of Focal Enhancement in Intracranial Aneurysms as a Surrogate Marker for Aneurysm Instability. Neuroradiology.

[12] Peng F., Xia J., Zhang F., Lu S., Wang H., Li J., Liu X., Zhong Y., Guo J., Duan Y. (2025). Intracranial Aneurysm Instability Prediction Model Based on 4D-Flow MRI and HR-MRI. Neurotherapeutics.

[13] Ishii D., Nakagawa D., Zanaty M., Roa J.A., Al Kasab S., Shaban A., Hudson J.S., Osorno-Cruz C., Byer S., Allan L. (2020). Quantitative Susceptibility Mapping and Vessel Wall Imaging as Screening Tools to Detect Microbleed in Sentinel Headache. J. Clin. Med..

[14] Wiebers D.O. (2003). Unruptured Intracranial Aneurysms: Natural History, Clinical Outcome, and Risks of Surgical and Endovascular Treatment. Lancet.

[15] Korja M., Lehto H., Juvela S. (2014). Lifelong Rupture Risk of Intracranial Aneurysms Depends on Risk Factors: A Prospective Finnish Cohort Study. Stroke.

[16] Kailaya-Vasan A., Frantzias J., Kailaya-Vasan J., Anderson I.A., Walsh D.C. (2022). Current Decision Support Tools Fail to Agree or Predict Therapeutic Decisions in a Single Cohort of Unruptured Intracranial Aneurysms. Acta Neurochir..

[17] Pettersson S.D., Skrzypkowska P., Pietrzak K., Och A., Siedlecki K., Czapla-Iskrzycka A., Klepinowski T., Fodor T., Filo J., Meyer-Szary J. (2024). Evaluation of PHASES Score for Predicting Rupture of Intracranial Aneurysms: Significance of Aneurysm Size. World Neurosurg..

[18] Villablanca J.P., Duckwiler G.R., Jahan R., Tateshima S., Martin N.A., Frazee J., Gonzalez N.R., Sayre J., Vinuela F.V. (2013). Natural History of Asymptomatic Unruptured Cerebral Aneurysms Evaluated at CT Angiography: Growth and Rupture Incidence and Correlation with Epidemiologic Risk Factors. Radiology.

[19] Matsubara S., Hadeishi H., Suzuki A., Yasui N., Nishimura H. (2004). Incidence and Risk Factors for the Growth of Unruptured Cerebral Aneurysms: Observation Using Serial Computerized Tomography Angiography. J. Neurosurg..

[20] Fujimura S., Yamanaka Y., Takao H., Ishibashi T., Otani K., Karagiozov K., Fukudome K., Yamamoto M., Murayama Y. (2024). Hemodynamic and Morphological Differences in Cerebral Aneurysms between before and after Rupture. J. Neurosurg..

[21] Laurence D.W., Homburg H., Yan F., Tang Q., Fung K.-M., Bohnstedt B.N., Holzapfel G.A., Lee C.-H. (2021). A Pilot Study on Biaxial Mechanical, Collagen Microstructural, and Morphological Characterizations of a Resected Human Intracranial Aneurysm Tissue. Sci. Rep..

[22] Frösen J., Piippo A., Paetau A., Kangasniemi M., Niemelä M., Hernesniemi J., Jääskeläinen J. (2004). Remodeling of Saccular Cerebral Artery Aneurysm Wall Is Associated With Rupture: Histological Analysis of 24 Unruptured and 42 Ruptured Cases. Stroke.

[23] Shimonaga K., Matsushige T., Ishii D., Sakamoto S., Hosogai M., Kawasumi T., Kaneko M., Ono C., Kurisu K. (2018). Clinicopathological Insights From Vessel Wall Imaging of Unruptured Intracranial Aneurysms. Stroke.

[24] Zhu C., Haraldsson H., Tian B., Meisel K., Ko N., Lawton M., Grinstead J., Ahn S., Laub G., Hess C. (2016). High Resolution Imaging of the Intracranial Vessel Wall at 3 and 7 T Using 3D Fast Spin Echo MRI. Magn. Reson. Mater. Phys. Biol. Med..

[25] Vranic J.E., Hartman J.B., Mossa-Basha M. (2021). High-Resolution Magnetic Resonance Vessel Wall Imaging for the Evaluation of Intracranial Vascular Pathology. Neuroimaging Clin. N. Am..

[26] Edjlali M., Gentric J.-C., Régent-Rodriguez C., Trystram D., Hassen W.B., Lion S., Nataf F., Raymond J., Wieben O., Turski P. (2014). Does Aneurysmal Wall Enhancement on Vessel Wall MRI Help to Distinguish Stable From Unstable Intracranial Aneurysms?. Stroke.

[27] Samaniego E.A., Roa J.A., Hasan D. (2019). Vessel Wall Imaging in Intracranial Aneurysms. J. NeuroInterventional Surg..

[28] Larsen N., Von Der Brelie C., Trick D., Riedel C.H., Lindner T., Madjidyar J., Jansen O., Synowitz M., Flüh C. (2018). Vessel Wall Enhancement in Unruptured Intracranial Aneurysms: An Indicator for Higher Risk of Rupture? High-Resolution MR Imaging and Correlated Histologic Findings. AJNR Am. J. Neuroradiol..

[29] Texakalidis P., Hilditch C.A., Lehman V., Lanzino G., Pereira V.M., Brinjikji W. (2018). Vessel Wall Imaging of Intracranial Aneurysms: Systematic Review and Meta-Analysis. World Neurosurg..

[30] Kleinloog R., Korkmaz E., Zwanenburg J.J.M., Kuijf H.J., Visser F., Blankena R., Post J.A., Ruigrok Y.M., Luijten P.R., Regli L. (2014). Visualization of the Aneurysm Wall: A 7.0-Tesla Magnetic Resonance Imaging Study. Neurosurgery.

[31] Blankena R., Kleinloog R., Verweij B.H., van Ooij P., Haken B.T., Luijten P.R., Rinkel G.J.E., Zwanenburg J.J.M. (2016). Thinner Regions of Intracranial Aneurysm Wall Correlate with Regions of Higher Wall Shear Stress: A 7T MRI Study. Am. J. Neuroradiol..

[32] Omodaka S., Endo H., Niizuma K., Fujimura M., Inoue T., Sato K., Sugiyama S.-I., Tominaga T. (2016). Quantitative Assessment of Circumferential Enhancement along the Wall of Cerebral Aneurysms Using MR Imaging. AJNR Am. J. Neuroradiol..

[33] Raghuram A., Varon A., Roa J.A., Ishii D., Lu Y., Raghavan M.L., Wu C., Magnotta V.A., Hasan D.M., Koscik T.R. (2021). Semiautomated 3D Mapping of Aneurysmal Wall Enhancement with 7T-MRI. Sci. Rep..

[34] Mandell D.M., Mossa-Basha M., Qiao Y., Hess C.P., Hui F., Matouk C., Johnson M.H., Daemen M.J.A.P., Vossough A., Edjlali M. (2017). Intracranial Vessel Wall MRI: Principles and Expert Consensus Recommendations of the American Society of Neuroradiology. AJNR Am. J. Neuroradiol..

[35] Feng J., Liu X., Zhang Z., Wu Y., Li Z., Zhang Q., Jiang Y., You W., Liu P., Wang Y. (2022). Comparison of 7 T and 3 T Vessel Wall MRI for the Evaluation of Intracranial Aneurysm Wall. Eur. Radiol..

[36] Schnell S., Ansari S.A., Vakil P., Wasielewski M., Carr M.L., Hurley M.C., Bendok B.R., Batjer H., Carroll T.J., Carr J. (2014). Three-Dimensional Hemodynamics in Intracranial Aneurysms: Influence of Size and Morphology. J. Magn. Reson. Imaging.

[37] Schnell S., Wu C., Ansari S.A. (2016). 4D MRI Flow Examinations in Cerebral and Extracerebral Vessels. Ready for Clinical Routine?. Curr. Opin. Neurol..

[38] Cebral J.R., Castro M.A., Burgess J.E., Pergolizzi R.S., Sheridan M.J., Putman C.M. (2005). Characterization of Cerebral Aneurysms for Assessing Risk of Rupture by Using Patient-Specific Computational Hemodynamics Models. AJNR Am. J. Neuroradiol..

[39] Zhuang B., Sirajuddin A., Zhao S., Lu M. (2021). The Role of 4D Flow MRI for Clinical Applications in Cardiovascular Disease: Current Status and Future Perspectives. Quant. Imaging Med. Surg..

[40] van Tuijl R.J., den Hertog C.S., Timmins K.M., Velthuis B.K., van Ooij P., Zwanenburg J.J.M., Ruigrok Y.M., Schaaf I.C. (2024). van der Intra-Aneurysmal High-Resolution 4D MR Flow Imaging for Hemodynamic Imaging Markers in Intracranial Aneurysm Instability. Am. J. Neuroradiol..

[41] Huang F., Janiga G., Berg P., Hosseini S.A. (2024). On Flow Fluctuations in Ruptured and Unruptured Intracranial Aneurysms: Resolved Numerical Study. Sci. Rep..

[42] Gottwald L.M., Töger J., Markenroth Bloch K., Peper E.S., Coolen B.F., Strijkers G.J., van Ooij P., Nederveen A.J. (2020). High Spatiotemporal Resolution 4D Flow MRI of Intracranial Aneurysms at 7T in 10 Minutes. AJNR Am. J. Neuroradiol..

[43] Li Y., Amili O., Moen S., Van de Moortele P.-F., Grande A., Jagadeesan B., Coletti F. (2022). Flow Residence Time in Intracranial Aneurysms Evaluated by in Vitro 4D Flow MRI. J. Biomech..

[44] Sugiyama S., Niizuma K., Nakayama T., Shimizu H., Endo H., Inoue T., Fujimura M., Ohta M., Takahashi A., Tominaga T. (2013). Relative Residence Time Prolongation in Intracranial Aneurysms: A Possible Association with Atherosclerosis. Neurosurgery.

[45] Bakhshinejad A., Baghaie A., Vali A., Saloner D., Rayz V.L., D’Souza R.M. (2017). Merging Computational Fluid Dynamics and 4D Flow MRI Using Proper Orthogonal Decomposition and Ridge Regression. J. Biomech..

[46] van Ooij P., Guédon A., Poelma C., Schneiders J., Rutten M.C.M., Marquering H.A., Majoie C.B., VanBavel E., Nederveen A.J. (2012). Complex Flow Patterns in a Real-Size Intracranial Aneurysm Phantom: Phase Contrast MRI Compared with Particle Image Velocimetry and Computational Fluid Dynamics. NMR Biomed..

[47] Haacke E.M., Liu S., Buch S., Zheng W., Wu D., Ye Y. (2015). Quantitative Susceptibility Mapping: Current Status and Future Directions. Magn. Reson. Imaging.

[48] Park S.I., Kim D., Jung S.C., Nam Y., Alabdulwahhab A., Lee J., Choi K.M. (2023). Feasibility and Intra-and Interobserver Reproducibility of Quantitative Susceptibility Mapping with Radiomic Features for Intracranial Dissecting Intramural Hematomas and Atherosclerotic Calcifications. Sci. Rep..

[49] Madden D.J., Merenstein J.L. (2023). Quantitative Susceptibility Mapping of Brain Iron in Healthy Aging and Cognition. Neuroimage.

[50] Chen H., Yang A., Huang W., Du L., Liu B., Lv K., Luan J., Hu P., Shmuel A., Shu N. (2024). Associations of Quantitative Susceptibility Mapping with Cortical Atrophy and Brain Connectome in Alzheimer’s Disease: A Multi-Parametric Study. Neuroimage.

[51] Aimo A., Huang L., Tyler A., Barison A., Martini N., Saccaro L.F., Roujol S., Masci P.-G. (2022). Quantitative Susceptibility Mapping (QSM) of the Cardiovascular System: Challenges and Perspectives. J. Cardiovasc. Magn. Reson..

[52] Nakagawa D., Cushing C., Nagahama Y., Allan L., Hasan D. (2017). Quantitative Susceptibility Mapping as a Possible Tool to Radiographically Diagnose Sentinel Headache Associated with Intracranial Aneurysm: Case Report. World Neurosurg..

[53] Nakagawa D., Kudo K., Awe O., Zanaty M., Nagahama Y., Cushing C., Magnotta V., Hayakawa M., Allan L., Greenlee J. (2019). Detection of Microbleeds Associated with Sentinel Headache Using MRI Quantitative Susceptibility Mapping: Pilot Study. J. Neurosurg..

[54] Park A., Chute C., Rajpurkar P., Lou J., Ball R.L., Shpanskaya K., Jabarkheel R., Kim L.H., McKenna E., Tseng J. (2019). Deep Learning-Assisted Diagnosis of Cerebral Aneurysms Using the HeadXNet Model. JAMA Netw. Open.

[55] Sichtermann T., Faron A., Sijben R., Teichert N., Freiherr J., Wiesmann M. (2019). Deep Learning-Based Detection of Intracranial Aneurysms in 3D TOF-MRA. AJNR Am. J. Neuroradiol..

[56] Liu J., Chen Y., Lan L., Lin B., Chen W., Wang M., Li R., Yang Y., Zhao B., Hu Z. (2018). Prediction of Rupture Risk in Anterior Communicating Artery Aneurysms with a Feed-Forward Artificial Neural Network. Eur. Radiol..

[57] Wen Z., Wang Y., Zhong Y., Hu Y., Yang C., Peng Y., Zhan X., Zhou P., Zeng Z. (2024). Advances in Research and Application of Artificial Intelligence and Radiomic Predictive Models Based on Intracranial Aneurysm Images. Front. Neurol..

[58] Peper E.S., van Ooij P., Jung B., Huber A., Gräni C., Bastiaansen J.A.M. (2022). Advances in Machine Learning Applications for Cardiovascular 4D Flow MRI. Front. Cardiovasc. Med..

